# Visceral adiposity index, fitness and clustered cardiovascular disease risk in adolescents

**DOI:** 10.4102/phcfm.v16i1.4474

**Published:** 2024-06-29

**Authors:** Danladi I. Musa, Daniel T. Goon, Rafiu O. Okuneye, Mary O. Onoja-Alexander, Joseph I. Momoh, Tessy O. Angba

**Affiliations:** 1 Department of Human Kinetics and Health Education, Faculty of Education, Kogi State University, Anyigba, Nigeria; 2 Faculty of Health Sciences, University of Limpopo, Sovenga, South Africa; 3 Department of Human Kinetics, Sports and Health Education, Faculty of Education, Lagos State University, Lagos, Nigeria; 4 Department of Community Medicine, Faculty of Health Sciences, Kogi State University, Anyigba, Nigeria; 5 Department of Human Physiology, Faculty of Basic Medical Sciences, Kogi State University, Anyigba, Nigeria; 6 Department of Human Kinetics, Faculty of Education, National Open University, Abuja, Nigeria

**Keywords:** adolescents, abdominal adiposity, fitness, cardiovascular health, primary prevention

## Abstract

**Background:**

Clustering of cardiovascular disease (CVD) risk factors have been observed in children and adolescents, but its association with visceral adiposity index (VAI) and cardiorespiratory fitness (CRF) in adolescents has rarely been studied.

**Aim:**

This study determines the independent associations of VAI and CRF with the clustering of cardiovascular disease risk (CVDr) among Nigerian adolescents.

**Setting:**

Adolescents from specific secondary schools in Kogi East, North Central Nigeria participated in the study.

**Methods:**

A cross-sectional sample of 403 adolescents (202 boys and 201 girls) aged 11 years – 19 years were evaluated for VAI, CRF and CVDr. Using identified risk factors, a clustered CVDr score was generated. The association between VAI, CRF and clustered CVDr was evaluated using regression models that controlled for age, gender and maturity status.

**Results:**

Fitness was negatively associated with CVDr (*β* = -0.268, *p* < 0.001), while VAI was positively correlated with CVDr (*β* = 0.379, *p* < 0.001). After CRF or VAI adjustment, the independent association with the dependent variable remained significant. The odds of an adolescent with elevated VAI being at risk of CVD was 4.7 times higher than his peers. Unfit adolescents were 2.1 times more likely to develop CVDr.

**Conclusion:**

Both VAI and CRF were independently associated with the clustering of CVDr in Nigerian adolescents. The findings suggest that health promotion efforts focusing on healthy diet and aerobic-type physical activity programmes should be encouraged among the youth to reduce the risk of CVD.

**Contribution:**

This study shows that improving visceral adipose tissue and fitness may lower CVD risk factors in adolescents, which is significant for public health.

## Introduction

Cardiovascular disease (CVD) is widely recognised as the major cause of disability and mortality among adults worldwide.^1^ The World Health Organization (WHO) reports that CVD contributed to 32% of all deaths worldwide in 2019; almost 75% of these deaths occurred in low- and middle-income nations.^2^ Although CVD is predominantly a problem for adults, it begins in childhood.^3,4^ Additionally, there is evidence of CVD and metabolic risk factor clustering in children and adolescents,^5,6^ which persists throughout adulthood.^7^ As a result, early identification of at-risk adolescents is crucial for primary prevention and a better health prospect. Considering the absence of commonly acknowledged diagnostic criteria for metabolic syndrome (MetS) in the youth population, a continuous MetS score has been proposed for youth research.^8^ This method has been used by many researchers.^5,6^

Primary prevention of CVD risk factors, as well as an understanding of their interconnections, are important strategies for reducing the epidemic. Blood lipid profile parameters, obesity and physical inactivity are all strong predictors of CVD in adults^9,10^ and youth.^5,11^ Several cross-sectional^11,12^ and prospective studies^13,14^ have shown that individuals with high levels of cardiorespiratory fitness (CRF) are at reduced risk of MetS, CVD and all-cause mortality.^1,15^ Recently, there has been increased focus on the function of visceral fat (VAT) in the development and promotion of a variety of health problems, including subclinical inflammation, insulin resistance and coronary atherosclerosis.^16,17^ The visceral adiposity index (VAI), which quantifies VAT dysfunction, exhibits a significant correlation with insulin resistance and cardiovascular events among the adult population.^18,19^ The VAI combines anthropometric measurements of body mass index (BMI) and waist circumference (WC) with blood lipid parameters, namely triglycerides (TG) and high-density lipoprotein cholesterol (HDL-C). The VAI is a recognised biomarker for noncommunicable diseases in adults, including MetS and CVD.^10,20^

Several studies have evaluated the relationships between physical activity or CRF and individual CVDs^21,22^ or the clustering of CVD risk scores^5,12,13^ in children and adolescents. These studies have demonstrated that higher levels of CRF are linked to a reduced occurrence of CVD clustering. However, there is hardly any study that examined the combined association of CRF and VAI with the risk of clustered CVD in adolescents to date. Weight disorders, especially overweight (OW) and obesity, are major aetiological factors for increased cardiovascular disease risk (CVDr) in adolescents.^7,21^ Despite the clinical importance of VAI and its co-morbidities in adults, there is limited information on the influence of this biomarker of cardiometabolic disease on cardiovascular health in adolescents. Furthermore, there has been little research into the relationship among VAI, CRF and CVD risk, particularly among adolescents. This study has three goals. Firstly, this study examines the independent and joint associations of VAI and CRF with clustered CVD in North-Central Nigerian adolescents. Secondly, it characterised participants’ CVD risk profiles using a clustered CVD risk score. Finally, the study determined the values of the independent variables that indicate a clustering of CVD risk among participants. Information on the interrelationships between these variables may serve as key public health promotion targets in the prevention and management of CVD in adolescents.

## Research methods and design

### Study setting and design

From selected secondary schools in Kogi East, North Central Nigeria, a cross-sectional study was undertaken involving schoolboys and girls aged 11 years to 19 years, who were early maturers based on age at peak height velocity. The data collection timeframe for the project lasted from September to December 2019.

### Study population and sampling strategy

The study participants comprised 418 adolescents who were randomly selected across four secondary schools from the study area using the adjusted Taro Yamani sample size determination formula.^24^ The calculation resulted in 380 participants as the minimum size, which was increased to 403 participants to improve representativeness, and to account for possible attrition usually associated with data collection. A comprehensive description of the study setting, eligibility criteria, sampling technique and pilot test had been described previously.^21^ Once appropriate authorisation from the heads of the participating schools was obtained, the participants were given a detailed explanation of the study’s goals and testing protocols.

### Data collection procedure

The testing team visited the participating schools twice to collect data. During the first visit, physical attributes and fitness were measured, while the final appointment was for the measurement of clinical and biochemical parameters. To ensure consistency, all tests were performed sequentially by the same members of the testing team throughout the project.

### Physical and physiological measurements

Participants’ physical characteristics were assessed in accordance with the International Society for the Advancement of Kinanthropometry (ISAK) procedures.^25^ Stature, body mass, percentage of body fat, WC and BMI were assessed using anthropometric methods.^21^ Participants were grouped as healthy weight (HW) and OW based on their BMI values.^26^ Sexual maturity was estimated from chronological age and stature using Moore et al.’s prediction equation.^27^ Maturity off-set (MO) was estimated directly from the formula. The difference between MO and age determines APHV.

Resting systolic blood pressure (SBP) and diastolic blood pressure (DBP) were assessed after each participant sat quietly for 10 min with an automated device (HEM-705 CP; Omron, Tokyo, Japan). The data were collected three times, at a 2-min interval, and the mean value was used for analysis. The blood pressure cut-off for hypertension (HTN) was determined using an established standard.^28^

Cardiorespiratory fitness was assessed by the 20-m multistage shuttle run test (20-MST). The test is an aerobic capacity test with increasing intensity. Throughout the test, participants were encouraged verbally to run until volitional fatigue. The test is considered a valid predictor of peak oxygen uptake in youth.^29^ The number of laps or shuttles completed by each participant was used to estimate CRF.^29^ The test’s specific administrative procedures and participant classification into fitness categories have been published.^26^

### Clinical and Biochemical measurement

Fasting plasma total cholesterol (TCL), low-density lipoprotein cholesterol (LDL-C), HDL-C, TG and fasting plasma glucose (FPG) were measured from capillary blood samples between 9:00 and 11:00. using the Cardio-Check Plus Analyzer (CCPA) (PTS Diagnostics, Indianapolis, IN, USA).^21^ Two qualified nurses and a laboratory technologist performed the test. Participants were given a 10-min rest period before taking their turns for the test. The protocol’s details were previously described.^21^ The CCPA measures blood lipids accurately.^30^ Visceral adiposity index was calculated using the formula of Amato and Colleagues^19^:


$$\begin{array}{l} \text{VAI }(\text{girls})=\left(\text{WC}/39.58+[1.89\times \text{BMI}]\right)\\ \;\times (\text{TG}/0.81)\times (1.52/\text{HDL-C}) \end{array}$$ [Eqn 1]

$$\begin{array}{l} \text{VAI }(\text{boys})=\left(\text{WC}/39.68+[1.88\times \text{BMI}]\right)\\ \;\times (\text{TG}/1.03)\times (1.31/\text{HDL-C}) \end{array}$$ [Eqn 2]

where, WC was expressed in centimetres, BMI in kg.m^−2^, HDL-C and TG in mMol.L^−2^.

### Clustered cardiovascular disease risk score

While it is difficult to identify variations between individual risk factors among youth,^8^ a clustered CVDr score was derived by summing the sex-standardised residuals (Z-scores) for SBP, DBP, TCL, LDL-C, HDL-C (inverted) and TG. A lower clustered CVDr score is suggestive of a more favourable risk factor profile. This method has been employed in a variety of youth research.^23,31^ Participants with a CVDr score +1SD higher than the overall mean were deemed to be at risk of clustered CVD.^32^

We assessed CVDr abnormalities using the International Diabetes Federation (IDF) criteria^33^: TG (≥ 1.7 mMol.L^−1^), HDL-C (≤ 1.04 mMol.L^−1^) and FPG (≥ 5.6 mMol.L^−1^). Adolescents who had one or more risks were categorised as ‘at risk’, while those who had none were classified as ‘no risk’. As there is no defined cut-off value for VAI that separates normal from dysfunctional visceral adiposity in the paediatric population, the sample was divided into tertiles, with the lowest value of the highest tertile serving as the threshold.^33^ The sample’s benchmark was 1.1 (girls = 1.35; boys = 0.83). This method is regarded as a useful estimate of the VAI benchmark and has been employed by various scholars.^18,34^

### Data analysis

Means, standard deviations, frequencies and percentages were used to represent descriptive data. The normality of distribution was checked for all variables using the Kolmogorov–Smirnov test. Because of absenteeism and incomplete data, 403 out of 418 adolescents completed the measurements. Therefore, data for all variables were available for 403 participants that were used in statistical analysis. This amounted to a participation rate of 96%. Significant differences between genders on all study variables were determined with the independent samples *t*-test or, where appropriate, the Mann–Whitney U test. Because there was no significant interaction between age, biological maturity and gender, all analyses were performed with both sexes pooled to enhance statistical power. Bivariate analyses were performed to examine partial correlations among the CVDr, VAI and CRF. The relationships between VAI and CRF, as well as their relative importance, were examined using multivariate regression models while adjusting for sex, age and sexual maturation. The logistic regression model assessed the independent association of VAI and CRF with CVDr. The independent variables’ predictive performance was examined using receiver operating characteristic curve analysis (ROC), with a 95% confidence interval (95%CI). Values of the area under the curve (AUC), sensitivity and specificity were used to identify CVDr benchmarks. The AUC values were interpreted using Swets’ technique.^35^ The Statistical Package for Social Sciences (SPSS) version 20 was utilised for analyses, with *p*-values ≤ 0.05 deemed statistically significant.

### Ethical considerations

The study protocol was approved by the Ethical Review Committee of the College of Health Sciences, Kogi State University, Nigeria (Ref. No. COHS/02/25/2020). Parents or guardians and participants provided written informed consent and assent, respectively, prior to data collection. The Declaration of Helsinki was adhered to for ethical guidelines.

## Results

**FIGURE 1:**
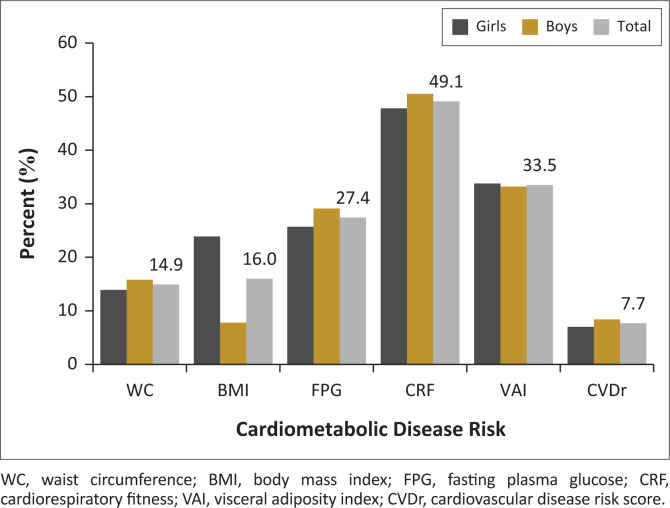
Participants’ cardiometabolic disease risk profile stratified by sex.

Table 1 displays the participants’ general characteristics. Girls had greater BMI (*p* < 0.001), body mass (*p* = 0.016), MO (*p* < 0.001) and WC (*p* = 0.001) compared to boys. Age, stature, FPG, DBP, TG and HDL-C were similar between sexes (*p* > 0.05). Boys had more favourable SBP (*p* = 0.016), TC (*p* = 0.028), LDL-C (*p* = 0.028), the independent variables (*p* < 0.001) and the dependent variable (*p* = 0.005) compared to girls. Participants’ CVD risk profiles are presented in Table 2. Aside from the TC and LDL-C, which had similar characteristics for both boys and girls, all other risk factors differed significantly across sexes, with girls being more vulnerable than boys. In terms of the proportion of participants at risk for different health parameters, boys were more vulnerable (Figure 1). In general, a preponderance of adolescents was at risk of low fitness (49.1%), followed by elevated VAI (33.5%).

**TABLE 1:** General characteristics of participants stratified by gender (*N* = 403).

Variable	Total (*N* = 403) Mean ± s.d.	Girls (*N* = 201) Mean ± s.d.	Boys (*N* = 202) Mean ± s.d.	*t*-value	*p*
Age (years)	14.7 ± 2.3	14.8 ± 2.3	14.7 ± 2.2	0.586	0.558
AHPV (years)	13.3 ± 1.1	12.5 ± 0.8	14.1 ± 0.8	20.068	< 0.001
MO (years)	1.4 ± 1.3	2.3 ± 1.7	0.6 ± 1.0	10.029	< 0.001
Height (cm)	160.2 ± 9.8	159.6 ± 7.1	160.9 ± 11.9	0.303	0.193
Weight (kg)	53.1 ± 12.5	55.5 ±12.1	50.8 ± 12.5	3.784	0.016
BMI (kg.m^−2^)	20.5 ± 3.5	21.7 ± 4.0	19.3 ± 2.6	6.929	< 0.001
WC (cm)	65.8 ± 8.8	67.2 ± 9.4	64.4 ± 8.0	2.273	0.001
TG (mMol.L^−1^)	1.0 ± 0.9	1.1 ± 1.2	0.9 ± 0.4	1.653	0.100
HDL-C (mMol.L^−1^)	1.3 ± 0.4	1.3 ± 0.3	1.3 ± 0.4	1.090	0.276
LDL-C (mMol.L^−1^)	2.2 ± 0.7	2.2 ± 0.6	2.1 ± 0.7	2.205	0.028
TC (mMol.L^−1^)	3.5 ± 0.8	1.1 ± 1.2	0.9 ± 0.4	2.211	0.028
20-MST (lap)	32.2 ± 16.8	24.9 ± 13.3	39.5 ± 16.8	9.697	< 0.001
VAI	1.2 ± 0.7	1.6 ± 0.8	0.8 ± 0.6	3.912	< 0.001
SBP (mmHg)	105.6 ± 16.6	107.8 ± 16.7	103.5 ± 16.2	2.587	0.010
DBP (mmHg)	69.9 ± 14.5	70.4 ± 15.3	69.5 ± 13.5	0.595	0.552
FBG (mMol.L^−1^)	5.1 ± 0.7	5.0 ± 0.7	5.1 ± 0.7	0.401	0.157
CVDr	−7.7 ± 3.1	−7.2 ±3.2	−8.1 ± 3.0	2.839	0.005

APHV, age at peak height velocity; MO, maturity off-set; BMI, body mass index; WC, waist circumference; TG, triglycerides; HDL-C, high-density lipoprotein cholesterol; LDL-C, low-density lipoprotein cholesterol; TC, total cholesterol; 20-MST, multistage shuttle run test; VAI, visceral adiposity index; SBP, systolic blood pressure; DBP, diastolic blood pressure; FBG, fasting blood glucose; CVDr, cardiovascular disease risk score; s.d., standard deviation.

**TABLE 2:** Comparison of participants according to level of cardiovascular disease risk (*N* = 403).

Variable	Girls (*N* = 201) Mean ± s.d.		Boys (*N* = 202) Mean ± s.d.	
	Low (*N* = 92)	High (*N* = 109)	Low (*N* = 99)	High (*N* = 103)
Age (years)	13.8 ± 2.5	15.6 ± 1.8**	13.6 ± 1.8	15.7 ± 2.1**
BMI (kg.m^−2^)	20.9 ± 4.1	22.2 ± 3.7*	18.7 ± 2.1	20.0 ± 2.9**
WC (cm)	63.1 ± 6.9	70.7 ± 9.8**	59.4 ± 3.8	69.1 ± 8.1**
20-MST (lap)	29.3 ± 13.0	21.2 ± 12.4**	47.2 ± 4.4	32.1 ± 15.7**
VAI	0.8 ± 0.3	2.3 ± 1.6**	0.6 ± 0.2	1.1 ± 0.7**
FBG (mMol.L^−1^)	4.8 ± 0.6	5.2 ± 0.8**	4.8 ± 0.4	5.4 ± 0.8**
TC (mMol.L^−1^)	3.6 ± 0.9	3.6 ± 0.8	3.5 ± 0.7	3.3 ± 0.7
HDL-C (mMol.L^−1^)	1.4 ± 0.2	1.1 ± 0.2**	1.5 ± 0.5	1.2 ± 0.4**
LDL-C (mMol.L^−1^)	2.2 ± 0.5	2.2 ± 0.7	2.0 ± 0.5	2.2 ± 0.9
TG (mMol.L^−1^)	0.7 ± 0.3	1.3 ± 0.6**	0.8 ± 0.3	1.0 ± 0.4**
SBP (mmHg)	101.0 ± 15.3	113.4 ± 15.6**	96.8.1 ± 14.7	100.0 ± 15.0**
DBP (mmHg)	63.7 ± 12.3	75.9 ± 15.5**	63.7 ± 10.4	75.1 ± 13.9**
CVDr	−8.9 ± 2.5	−5.8 ± 3.0**	−9.6 ± 2.1	−6.6 ± 2.9**

BMI, body mass index; WC, waist circumference; TG, triglycerides; HDL-C, high-density lipoprotein cholesterol; LDL-C, low-density lipoprotein cholesterol; TC, total cholesterol; 20-MST, multistage shuttle run test; VAI, visceral adiposity index; SBP, systolic blood pressure; DBP, diastolic blood pressure; FBG, fasting blood glucose; CVDr, cardiovascular disease risk score.

** *p* < 0.001.

The multivariate regression models were adjusted for sex, age and maturity status (Table 3). The dependent variable was significantly associated with CRF (*r* = 0.241) and VAI (*r* = 0.398). The correlations were weak to moderate. Visceral adiposity index correlated positively with CVDr (*p* < 0.001), explaining 15% of the variance in CVD risk. A rise in VAI units could result in a mean increase of CVDr by 1.2. Cardiorespiratory fitness was negatively associated with the dependent variable (*p* < 0.001) and accounted for 5% of CVDr variation. Each unit (lap) increase in CRF was associated with an average decrease of 0.93 in CVDr. Despite additional adjustments in model 2 for either CRF or VAI, the independent association with CVDr remained statistically significant (*p* < 0.001).

**TABLE 3:** Standardised regression summary on the relationship among visceral adiposity index, cardiorespiratory fitness and clustered cardiovascular disease risk.

Models	CRF				VAI			
	*R* ^2^	*β*	*R*^2^∆	*p*	*R* ^2^	*β*	*R*^2^∆	*p*
Model 1	0.264	−0.268	0.189	< 0.001	0.255	0.379	0.180	< 0.001
Model 2	0.129	−0.300	0.054	< 0.001	0.222	0.394	0.146	< 0.001

∆, change; CRF, cardiorespiratory fitness; VAI, visceral adiposity index.

Results of the logistic regression model after adjustment for the covariates showed that independent variables displayed a significant effect, with VAI demonstrating a greater explanatory power. Visceral adiposity index (OR = 4.7, 95%CI = 2.80–7.78, *p* < 0.001) was positively associated with the dependent variable, while CRF (OR = 2.1, 95%CI = 1.23–3.59, *p* = 0.006) was negatively related with the dependent variable. The odds of CVD risk among adolescents with elevated VAI was 4.7 folds compared to peers with favourable VAI levels. Furthermore, the likelihood of an unfit adolescent being at risk of CVD was 2.1 times that of a fit counterpart.

**FIGURE 2:**
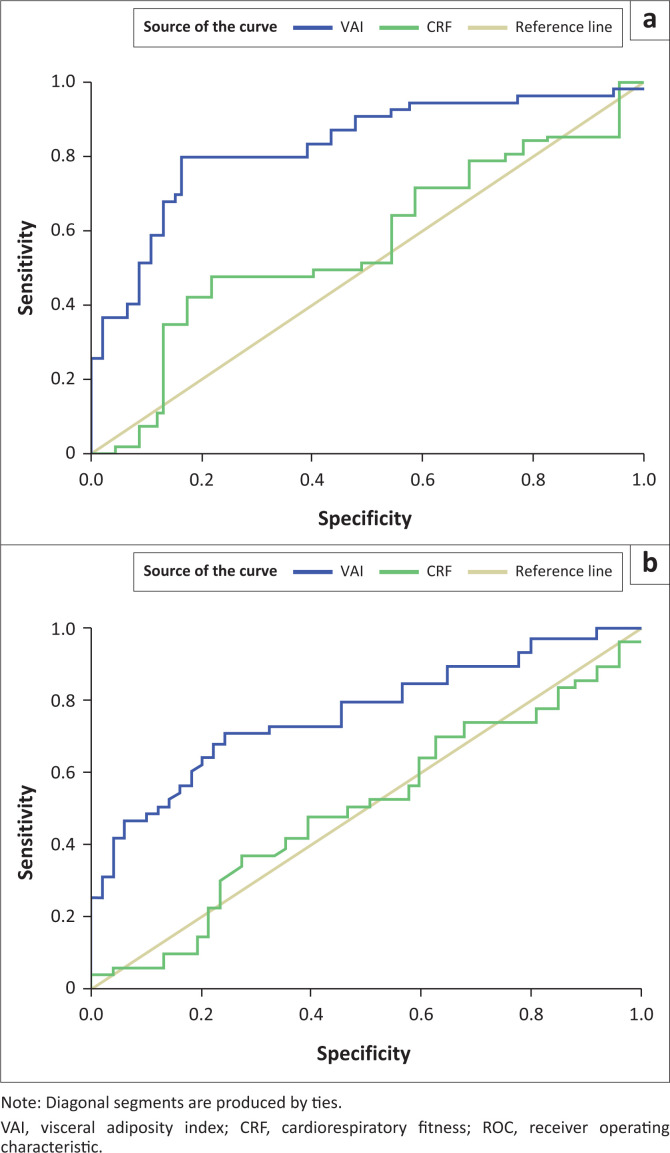
Sex-specific areas under the receiver operating characteristic curve of visceral adiposity index for (a) girls and (b) boys.

Results of the ROC analyses are presented in Table 4 and Figure 2. Only the AUC for VAI was significant in both sexes (*p* < 0.001), while that for CRF was not (*p* > 0.05). The VAI threshold for girls was 0.796 and that for boys was 0.570, with high sensitivity and low specificity.

**TABLE 4:** Receiver operating characteristic curve analysis for risk of clustered cardiorespiratory fitness among participants.

Gender	Variable	AUC	95%CI	Cut-point	Se	Sp	*p*
Girls	VAI	0.828	0.771–0.886	0.796	0.835	0.435	< 0.001
	CRF	0.564	0.484–0.645	24.500	0.569	0.543	0.117
Boys	VAI	0.764	0.699–0.829	0.570	0.796	0.455	< 0.001
	CRF	0.500	0.420–0.581	44.500	0.505	0.505	0.995

AUC, area under the curve; CRF, cardiorespiratory fitness; VAI, visceral adiposity index; Se, sensitivity; Sp, specificity; CI, confidence interval.

## Discussion

There is a growing body of evidence from studies in the western societies demonstrating that increased VAT and poor fitness may expose youth to CVDr.^36,37^ Although, CVD is becoming an increasingly important health issue in sub-Saharan Africa, even among young persons, the importance of visceral and subcutaneous fat tissues, as well as fitness in the development of CVD has received little attention in African adolescents, particularly in Nigeria. Key findings from this study include: CVD risk factor clustering exists among the study participants. Levels of VAI, CRF and other health indices were more favourable in adolescents with lower CVD risk. The independent variables and CVDr have weak to moderate relationships. Visceral adiposity index and CRF separately and jointly predicted CVDr, explaining 19% of the variance in the dependent variable, with VAI demonstrating greater explanatory power. The cut-off values for girls and boys of 0.796 and 0.570, respectively, are significantly lower than those documented for Caucasian and non-African youth.

This study’s findings demonstrated the existence of CVD risk factor clustering within this cohort of adolescents, with boys (8.4%) having a higher prevalence than girls (7.0%). Clustering has also been observed in different populations of youth globally.^14,23,38^ The overall prevalence of 7.7% recorded in this study appears to be higher than the reported rates of 5% and 5.4% for Iranian^39^ and Danish^38^ children, respectively. However, the prevalence rate of 11.9% among Norwegian children^23^ is substantially higher than that of the present study. Mean values for all CVD risk factors, apart from TCL and LDL-C are significantly more favourable in adolescents who had no risk of CVDr than in those who were at risk. This has been observed in previous research.^23^ Considering the vulnerability of participants to CVD risks, our study showed that, of the health indicators assessed, greater proportions of participants were at increased risk of low fitness and elevated VAI (Figure 1). These data demonstrate that visceral adipose tissue dysfunction and low fitness are more serious health issues than general or abdominal fat and plasma glucose abnormalities among the participants.

This study shows that both VAI and fitness independently predict clustered CVDr, with VAI displaying greater explanatory power. Earlier studies have reported similar findings with respect to VAI among children and adolescents^17,39^ showing significant associations between VAI and cardiometabolic disease risk factors in Asian youth and further concluding that VAI could be used as a surrogate marker of visceral adipose tissue and as a good indicator of MetS in the paediatric population.^17,39^ A study of obese Turkish girls documented similar findings.^40^ The contribution of VAI and fitness in predicting the dependent variable in the present study can be considered moderate (19%), and these results suggest that VAI is more important than fitness for predicting clustered CVDr in this cohort of adolescents. The present study provides evidence that improvement in both VAI and CRF could reduce CVD risk in youth and may be of public health significance. However, there is a caveat in the use of VAI in the paediatric population because its formula was derived from the BMI, WC, TG and HDL-C of Caucasian adults.^17,19^ In this study, VAI has demonstrated a unique association with clustered CVDr. Our findings therefore reinforce the applicability of VAI as an estimate of VAT dysfunction and cardiometabolic risk in adolescents, including those in Africa. Therefore, further studies in African children and adolescents are required to establish the validity of VAI in this population.

In our study, VAI was found to be a better predictor of the dependent variable than CRF. Indeed, the AUC for CRF was not significant in both sexes. These results suggest that visceral adipose tissue dysfunction may be a more serious health problem among adolescents. Visceral obesity is known to be associated with several co-morbidities including, increased adipocytokine production and proinflammatory biomarkers, HTN, dyslipidemia, decreased insulin sensitivity, increased risk of diabetes, atherosclerosis and mortality rates.^18,41^ It has also been documented that persons with higher levels of VAT also have correspondingly higher levels of inflammatory cytokines such as C-reactive proteins, interleukin-6 and tumour necrosis factor-α, all of which may result in the development of insulin resistance and metabolic abnormalities.^42^ Furthermore, fat cells around the abdomen release fat into blood vessels, thereby increasing the risk of MetS and CVD.^43,44^ Evidently, adipose tissue dysfunction is a serious health problem that requires intervention, even at the early stage of life. These results, therefore, highlight the need to encourage the consumption of a healthy diet and maintaining an active lifestyle, which could lead to achieving optimal cardiovascular health among adolescents.

Based on the findings from this study, it is plausible to assume that the association of increased visceral adipose tissue dysfunction and low fitness with the risk of CVD may be a result of unhealthy nutrition and sub-optimal physical activity.^22,42^ Furthermore, findings from this study suggest that having a lower VAI and a higher CRF might be important targets for maintaining and promoting cardiovascular health in youth.

One of the limitations of this study is the cross-sectional design that precludes the determination of causal association among the independent and dependent variables. In addition, the bulk of participants came from the Igala ethnic group of Kogi State, which is not representative of the multiracial Nigerian population. This obviously limits the generalisability of the study’s findings to other populations of youth in Nigeria. Therefore, a national population-based or multi-centre as well as longitudinal study is advocated. However, one key aspect of this study is the utilisation of ROC, which offered population-specific benchmarks for VAI in assessing participants’ CVD risk.

## Conclusion

Findings from this study demonstrated significant independent associations between VAI and CRF with CVDr in Nigerian adolescents, and the association persisted after adjusting for potential confounders. The combination of VAI and CRF in predicting the risk of CVD was weak to moderate. Thus, VAI and CRF could be explored as cost-effective and convenient tools for CVDr assessment in adolescents.
